# Genome Sequence of Mycobacteriophage Bassalto

**DOI:** 10.1128/mra.01351-22

**Published:** 2023-03-21

**Authors:** Nazir Barekzi, Meagan N. Wilkins, Aumon L. Williams, Afiya J. Moore, Zachary R. Duckett, Danielle M. Tindall, Donnetta R. Eaddy, Mary B. Johnson, Malcolm Bass, Catherine M. Mageeney

**Affiliations:** a Department of Biology, Norfolk State University, Norfolk, Virginia, USA; b Biotechnology and Bioengineering, Sandia National Laboratories, Livermore, California, USA; Queens College Department of Biology

## Abstract

Bassalto is a newly isolated phage of Mycobacterium smegmatis mc^2^155 from the campus grounds of Norfolk State University in Norfolk, VA. Bassalto belongs to the cluster B and subcluster B3 mycobacteriophages, based on the nucleotide composition and comparison to known mycobacteriophages.

## ANNOUNCEMENT

A significant number of Mycobacterium phage genomes have been annotated to understand their genomic structure, diversity, and evolution. Further study of phages contributes to the advancement of our general knowledge of genetics, biodiversity, and microbial ecology, which can be applied to environmental science and investigation of phage therapy to combat microbial pathogens such as Mycobacterium species (1).

Mycobacteriophage Bassalto was isolated from soil collected from the grounds of Norfolk State University in Norfolk, VA (36.8490817 N, 76.2593061 W), using the enriched isolation protocol in the Phage Discovery Guide (https://seaphagesphagediscoveryguide.helpdocsonline.com/home; accessed September 2017), with the host bacterium, Mycobacterium smegmatis mc^2^155. Phages were amplified using the web plate method and flooded with phage buffer followed by filtration (0.2-μm polytetrafluoroethylene [PTFE] VWR filter) of the supernatant to produce a high-titer lysate (HTL) containing greater than 10^9^ PFU/mL. Subsequently, phage DNA was extracted using the Promega Wizard DNA cleanup kit.

The genome of Bassalto was sequenced to at least 50× coverage using the Illumina MiSeq (2 × 250-bp) platform. Library preparation included tagmentation using the Illumina DNA prep tagmentation kit, followed by optimization with the unique dual index adapters from IDT for the Illumina Nextera DNA unique dual indexes (set C) kit. Sequencing yielded 242,627 total reads. The reads were trimmed using adapter trimming, and quality metrics were obtained using the Illumina FASTQ toolkit v1.1.0. The reads were assembled using Celera WGS v8.1 into a single 68,984-bp scaffold. Upon close inspection of the resulting scaffold, the assembled genome had three unresolved or unknown nucleotide blocks (N-blocks). To resolve the N-blocks, samples of the HTL were used with specific primers designed to flank each N-block by ~100 bp and PCR amplified (primers listed in Table 1). Subsequently, the DNA was run on an agarose gel; the fragment was extracted and sent out for sequencing by Genewiz, Inc.

**TABLE 1:** Primers used to resolve N-blocks

Primer name	Primer sequence (5′–3′)	Bassalto genome coordinates
BN1-up	TCGCCGAACGTCAGGCCGAGGAGGG	24741–24765
BN1-down	CCACCAGGGCGTGCGAGTCGTTG	25238–25216
B-N2-up	TCATGTCGCTGCCCGTAGG	37770–37788
B-N2-down	CCGAGTTCTTCATGACC	38153–38137
B-N3-up	CTCGTCAGCCGTGGCATCAC	38422–38441
B-N3-down	GTCGCGGCTGAGCACGTG	38546–38529

The complete resolved Bassalto genome was 69,113 bp with a G+C content of 67.5%. The coding DNA sequence predictions for Bassalto were determined using Web-based GeneMarkS (2) and command-line multiPhATE v0.5 (3) with the Glimmer v3.02 (4), Prodigal v2.6.3 (5), and PHANOTATE v0.13.0 (6) gene callers turned on. Open reading frames (ORFs) predicted using these gene callers were functionally annotated by conducting Web-based blastp (7) and HHpred (8) searches (using the databases PDB_mmCIF70 and Pfam-A_v35). The genome was run through Web-based tRNAscan-SE (9), and no tRNA genes were predicted. The genome had 99% nucleotide match to cluster B and subcluster B3 mycobacteriophages from the Actinobacteriophage Database, with similar sequence identity to Phaedrus, OrangeOswald, and Phyler (10), placing it in the class Caudoviricetes and genus *Pegunavirus*.

Bassalto contains 103 ORFs. There was a lack of defined ends, which is consistent among cluster B mycobacteriophages (11). In addition, the latter portion of Bassalto’s genome contains coding DNA sequences (CDSs) that encode proteins which function in DNA binding, host identification, and hydrolase inhibition. Notably, Bassalto contains two DNA polymerase genes (ORF95 and ORF98). There is also a notable region at the 3′ end of the genome (position 64899 to 65593) that has no nucleotide similarity to any sequence at NCBI and encodes a predicted HNH endonuclease.

### Data availability.

The Bassalto genome sequence is available at GenBank under accession number OP777409. The raw reads were deposited at the NCBI Sequence Read Archive (SRA) under BioProject accession number PRJNA931302.
